# Duration of post-vaccination immunity to yellow fever in volunteers ten years after a dose-response study – A complementary study

**DOI:** 10.1016/j.vaccine.2024.06.050

**Published:** 2024-10-24

**Authors:** Clarice Monteiro Vianna, Tatiana Guimarães Noronha, Luiz Antonio Bastos Camacho, Raissa Coelho Andrade, Ricardo Cristiano de Souza Brum, Eliane Matos dos Santos, Daniele Fernandes Aguiar, Maria Leticia Borges dos Santos, Robson Leite de Souza Cruz, Sheila Maria Barbosa de Lima, Adriana de Souza Azevedo Soares, Waleska Dias Schwarcz, Thalita da Matta de Castro, Janaína Reis Xavier, Deborah Araújo da Conceição, Akira Homma, Maria de Lourdes de Sousa Maia

**Affiliations:** a Instituto de Tecnologia em Imunobiológicos Bio-Manguinhos – FIOCRUZ, Rio de Janeiro, RJ, Brazil; b Universidade Federal Fluminense – UFF, Niterói, RJ, Brazil; c Escola Nacional de Saúde Pública – FIOCRUZ, Rio de Janeiro, RJ, Brazil

**Keywords:** Yellow fever, 17DD vaccine, Immunogenicity, Dose–response study

## Abstract

A single dose of standard yellow fever (YF) vaccine is considered to provide life-long protection. In this study, we evaluate the seropositivity conferred by lower doses 10 years post-vaccination. In 2009, Bio-Manguinhos/Fiocruz performed a dose–response study with the 17DD yellow fever vaccine, administering the vaccine in the usual mean dose of 27.476 IU and in decreasing doses (10.447 IU, 3.013 IU, 587 IU, 158 IU and 31 IU), with the usual volume and route (0,5 ml subcutaneous). The decreasing doses were obtained by dilution in the laboratory of the manufacturer and the lots in test had standard quality control and were produced by good manufacturing practices (GMP). Around 30 days after the vaccination, doses down to 587 IU had similar immunogenicity and the 158 IU and 31 IU were inferior to the full dose. The seropositivity was maintained for 10 months, except on the 31 IU group. Eight years after, 85 % of 318 participants evaluated in a follow-up, maintained seropositivity that was similar across groups. Consistently, antibody titers in the reduced-dose groups were also comparable to those of the full-dose group. The current study, 10 years later, showed similarity between the vaccine groups (six arms who received the YF vaccine in decreasing doses: 27.476 IU, 10.447 IU, 3.013 IU, 587 IU, 158 IU, 31 IU) both in relation of seropositivity and in the evaluation of the geometric mean titers. The seropositivity rates across subgroups were 83,1%, 90 %, 87 %, 93 %, 83,8% and 85 %, correspondingly. These findings provides further support to the long-term immunogenicity of lower doses.

**Clinical trial registry:** NCT04416477.

## Highlights

• A 2009 Bio-Manguinhos/Fiocruz YF vaccine dose–response study supported WHO's position for fractional dose for outbreaks.
• Part of the original cohort was reassessed for immunity duration and seroconversion.
• Seroconversion lasted 10 years.
• Reduced doses have shown effective seroconversion (>80 %).
• Antibody titers in reduced-dose *versus* full-dose groups remain equivalent.

## 1 Introduction

Yellow fever is a severe acute febrile infectious disease, transmitted by mosquitoes infected with a RNA Flavivirus that occurs in tropical and subtropical areas of Africa as well as in South and Central America. According to the transmission cycles the age, sex and occupation differ. The sylvatic (or jungle) cycle is most often in Central and South America. It affects non-human primates in forests by *Haemogogus* and *Aedes* mosquitoes. This disease occurs more often in young males and the age group most affected is above 15 years, due to greater exposure related to penetration into wild areas of the yellow fever endemicity areas. In Africa, the epidemiology is varied with sylvatic and domestic vector species in inter-human transmission. The urban cycle results when infected mosquitoes transmit the virus from person to person with large epidemics in densely populated areas where infected people move, and the local population has little or no immunity to yellow fever. *Aedes Aegypti* is the primary vector responsible for urban outbreaks in both Africa and South America [1], [2].

According to the World Health Organization (WHO) a single dose of yellow fever vaccine is sufficient to maintain lifelong protective immunity against yellow fever, therefore a booster dose is not necessary [3]. This issue is difficult to assess, and the interpretation of studies is complicated by different measures for seroprotective immunity [4]. Although studies indicate that the duration of protection after vaccination is long [5], there is a lot of evidence in literature that antibody titers drop over the years [6], reaching levels considered to be seronegative in at least a part of the vaccinated population [7]. This situation is more concerning for people who live in endemic areas, and who are exposed to the virus throughout their lives. Recent literature reviews and meta-analysis have showed the duration of immunity of yellow fever vaccine. Kling and colleagues in a systematic literature review and meta-analysis indicated six studies that addressed protection up to 10 years, with 88 % seroprotection rates in adults both endemic and non-endemic countries. After this period, immunity begins to diminish depending on the age and immune condition at the time of initial vaccination [8]. A recent systematic review and meta-analysis to assess 10 years or more long-protection after a single-dose of the vaccine found in healthy adult in non-endemic settings (mostly travelers) high rates of seroprotection (94 % vs 76 % in endemic settings) [9]. Until 2017, Brazil recommended revaccination once after 10 years in recommended areas or where there was a persisting epidemiological risk for travelers heading to those areas. Due to vaccine shortages in 2016 outbreaks in six countries, the Ministry of Health decided to adopt a single lifetime dose as recommended by the WHO [10].

Yellow fever occurs in sub-Saharan Africa, where it is endemic and intermittently epidemic. Urban outbreaks in Angola and the Democratic Republic of Congo in 2016, highlighted persistent challenges in eradicating yellow fever epidemics despite regular vaccination efforts [11]. In the Region of the Americas, yellow fever outbreaks have been reported in Brazil, Colombia and Peru. The number of confirmed and probable cases was the highest in the last ten years, and Brazil reported the start of a major epidemic in December 2016 [12]. With the intensification of the international mobility of people and climate change, the spread of infectious diseases such as yellow fever has become a global threat [13].

The YF vaccine has been considered the most relevant and effective prophylactic measure to prevent disease, inducing protective immunity within 10–30 days in approximately 95–99 % of primary adult vaccinated [1], [6], [7]. There are only four WHO pre-qualified manufactures, which supply YF vaccines for the Global Vaccine Action Plan, namely: Bio-Manguinhos/Fiocruz (Brazil), Sanofi Pasteur (France), Institut Pasteur de Dakar (Senegal), and the Chumakov Federal Scientific Center for Research & Development of Immune And Biological Products (Russian).

The minimum potency of the vaccine recommended by WHO should not be less than 1.000 IU/dose [14], [15]. As the potency of YF vaccines is usually much higher than the minimum needed to afford seroconversion, and currently available vaccines have achieved higher shelf stability, it seems reasonable to investigate the level and duration of the immune response to vaccine formulations with less viral particles. This would increase the supply of vaccines for routine vaccination and preparedness for future contingency.

In 2016, a group of yellow fever vaccine experts, concerned about the epidemiological situation of yellow fever in Africa, proposed the fractional use of yellow fever vaccine as a possible emergency solution [16]. The Strategic Advisory Group of Experts (SAGE) from WHO considered that the available evidence was sufficient to warrant the use of fractional doses (0,1 ml instead of 0,5 ml), as a safe and effective option for mass vaccination campaigns to control urban outbreaks in situations of YF vaccine shortage. The WHO has endorsed this recommendation [17]. Approximately 450 million doses per year are estimated to achieve high vaccination coverage (about 80 %) in areas of yellow fever viral circulation. At that time the annual production of YF vaccine accomplished only 80 million doses [18], [19]. In February 2018 in Brazil, after outbreaks a mass vaccination campaign was initiated using 1/5 (0,1 ml) of the standard dose [20].

WHO recommendations were mainly informed by a randomized dose–response study conducted by Bio-Manguinhos with the yellow fever vaccine produced by Bio-manguinhos/Fiocruz in 2009. The study tested 6 potencies of decreasing amounts of viral particles in 749 healthy young male adults from military units of the state of Rio de Janeiro, a non-endemic area. Six randomized subgroups received the YF vaccine in decreasing doses from 27.476 IU/dose (average dose for the vaccine lot used in the study) and dilutions of 10.447 IU, 3.013 IU, 587 IU, 158 IU and 31 IU/dose. The seroconversion was 97 % or higher with doses of 587 IU or above [21], and the duration of immunity after 10 months post-vaccination was also satisfactory for all participants who had seroconverted, except for the group that had received the lowest dose (31 IU) [21]. A follow-up study, using the same blood samples, also evaluating the production of interferon gamma and other cytokines, concluded that doses of 3.013 IU and above had equivalent responses to the standard vaccine of about 27.476 IU [22]. Based on these studies WHO position is that a fractional YF vaccine dose can be used as a part of an emergency response to an outbreak if there is a shortage of full-dose YF vaccine that exceeds the capacity of the global stockpile.

Eight years after vaccination, Martins et al (2018) followed up 271 participants who had seroconverted, were seropositive after 10 months, and had not been revaccinated. In recipients of doses from 587 IU to 27.476 IU seropositivity ranged from 80 % to 93 % [23].

In 2016, a yellow fever outbreak in Angola and the Democratic Republic of Congo (DRC) contribute to a global shortage of YF vaccine and the review by WHO to dose-sparing strategies for vaccination. A preemptive mass vaccination in 2016 in Kinshasa (capital city of DRC) used a fractional dose of the 17DD vaccine at one fifth (0,1 ml) of the standard dose. The results showed that serological results with the fractionated dose were like those reported with the full dose, in all age groups above 2 years and in both sexes [24]. Overall, 98 % seroconversion was observed. Between the end of 2017 and the beginning of 2018, in Uganda and Kenia, Juan-Giner and colleagues conducted a study with fractional doses of all WHO-prequalified yellow fever vaccines that showed no inferiority to the standard dose in inducing seroconversion 28 days after vaccination [25].

Considering the initial WHO position in 2013 for a single dose recommendation, and the use of fractional doses due to global shortages, it is relevant to study the long-term immunogenicity provided by the vaccines. The present study aims to evaluate the immune protection in a portion of the participants from the 2009-study 10 years later, considering the good results for seropositivity in the eight-year follow up.

## 2 Material and methods

### 2.1 Study design

This is a cross-sectional study, in healthy young male adults, military recruits, who received the first dose of yellow fever vaccine in 2009 as participants in the “Dose-Response Study of Yellow Fever Vaccine 17DD Produced by Bio-Manguinhos/Fiocruz” [21]. It is also an extension of the observational study conducted by Bio-Manguinhos in 2017 aiming to evaluate the duration of YF-specific humoral immunity after 8 years from first and unique YF vaccination [23]. Both studies supported the use of fractionated doses.

The present study like the study carried out in 2017, enrolled volunteers who participate of the dose–response study of 2009 [21], who were seronegative (negative PRNT levels) before the study vaccination and who were not revaccinated. Those who went on military missions, travelled, or lived in endemic areas were analyzed separately.

For recruitment, the databases with the information recorded by the studies carried out in 2009 and 2017 were used to contact participants by telephone or home visit. Same as 2017 study, application of study forms and blood collection took place at Fiocruz, at home or in a safe place, following verbal confirmation of the study participation in 2009, verification of YF revaccination after the 2009 study, and obtaining informed consent. Revaccination was double-checked: in the telephone contact and data collection in person. Participant’s enrolment and blood collection were held from April 2019 to August 2019, about 10 years after the dose–response study.

In this study, similar to the 2017 study, seeking to evaluate a possible interference of trips reported by research participants to the area of yellow fever vaccination recommendation since the dose–response study in 2009, an immunogenicity analysis was performed considering the presence and not of displacement to an area with recommendation of vaccination.

The immunogenicity analysis was performed by calculating the proportion of seropositivity (seropositive: ≥ 3.15 Log10 mIU/ml) and the geometric mean titer (GMT) per 2009 vaccine group.

The study was included in a clinical trial registry (NCT 04416477). The study protocol was approved by the Ethics Committee at Instituto Nacional de Infectologia Evandro Chagas, FIOCRUZ (Plataforma Brasil, CAEE – 0038.0.009.000–08). All procedures followed the Helsinki Declaration, the Brazilian ethical standards of scientific research involving human subjects and the good clinical practices.

### 2.2 Laboratory procedures

A volume of up to 10 ml of whole blood was collected (without anticoagulant), with a maximum of two blood collection attempts per volunteer. The material was taken to the Laboratory for Processing Biological Samples (LPAB), located at clinical site in Bio-Manguinhos/Fiocruz. The immunogenicity analysis allowed the calculation of the proportion of the seropositivity and the geometric mean titer (GMT) per 2009 vaccine group. Titration of neutralizing antibodies against YF was carried out with the micro Focus Reduction Neutralization Test (microFRNT) [26]. Samples were considered seropositive (reciprocal dilution ≥ 100) based on a ROC curve analysis (data not shown), comparing results obtained by classical PRNT and by FRNT following the conversion of the titers to International Units with cutoff point corresponding to 3.15 Log10 mIU/ml (Monkey international reference anti-YF serum – NIBISC) [27]. This allows the microFRNT results to be correlated with the classical PRNT.

Seropositive samples were calculated considering the YF antibody titer ≥ 1:100 (3.15 Log_10_ mIU/ml) as a cut-off point. Samples were considered seronegative with antibody titer ≤ 1:70 and those within 1:71 and 1:99 were classified as indeterminate (gray zone).

### 2.3 Statistical analysis

Statical analysis of neutralizing antibodies was done blindly, at first, by comparing the proportion of seropositivity across groups using chi-square or Fisher’s Tests, as indicated. Analysis of variance (ANOVA) of Log10 transformed titers was also performed. After completion of data analysis and, disclosure of codes, seroprotection rates and antibody neutralizing levels in recipients of lower vaccine doses were compared to those in the reference group (27.476 IU). For those who had travelled or lived in endemic areas, or went on military missions to endemic areas, there was a separate analysis, to account for putative “natural booster”. The geometric mean titers of each group were compared with the group that received the standard dose in the dose–response study, using Mann-Whitney test. Three levels of analysis were considered: participants adhering to the protocol; participants who, in addition to the above conditions, did not travel to yellow fever endemic areas after the dose–response study; all participants who provided blood samples in this study.

For statistical analysis, SPSS v.20.0, Stata v. 15, and WinPepi v. 11.54 were used. Antibody neutralizing levels are presented in mIU/mL, and the geometric mean titers are presented with 95 % CI.

## 3 Results

**Fig. 1:**
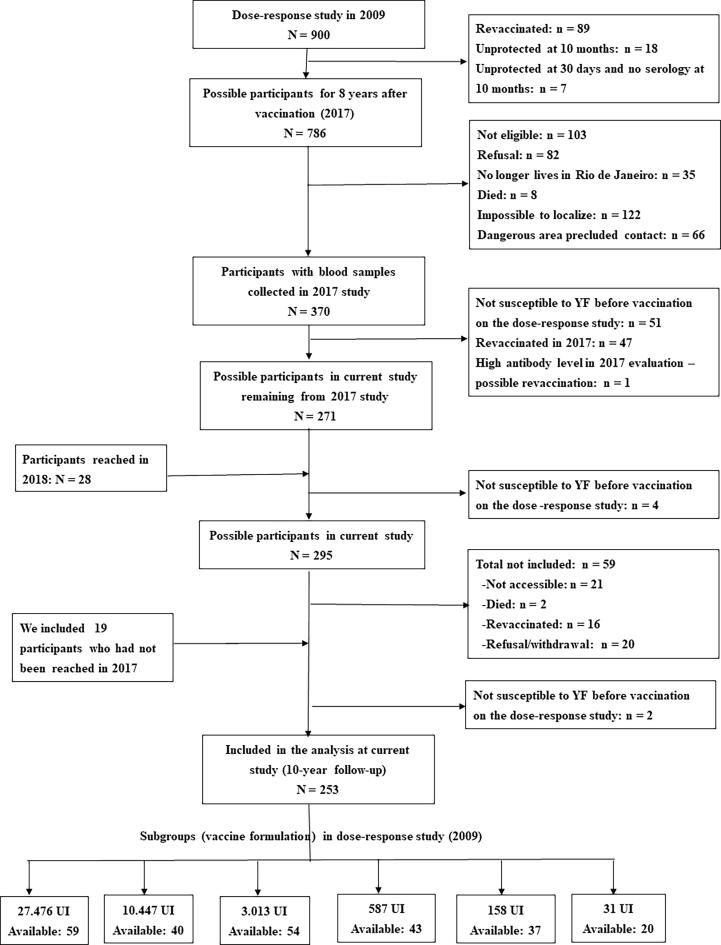
Formation of cohorts for verification of the serological status for yellow fever.

From the 900-volunteers original group enrolled in the dose–response study in 2009 after a persevering search, we were able to reach 253 eligible individuals as per criteria in Fig. 1 with also shows the dose subgroups they had been assigned to.

For these 253 research participants who adhered to the protocol, the proportion of seropositivity 10 years after vaccination ranged from 83.1 % to 93.0 % among vaccine groups but the difference was not statistically significant (p = 0.700). The differences in seropositivity between each reduced dose and the standard dose exhibited considerable variation without any discernible pattern (Table 1). Geometric mean titers showed substantial differences across groups, without statistical significance (p-value = 0.896) and no evident pattern in the GMT ratio between each reduced dose and the standard dose (Table 2).

**Table 1:** Proportion of seropositivity of participants on the dose–response study 10 years after vaccination, by vaccine group.

Group	Total	Seropositive participants*		Comparison to the reference vaccine	
	N	n	%	Difference of SP	95 % CI
27.476 IU	59	49	83.1	−	−
10.447 IU	40	36	90.0	6.9	(−6.4; 20.3)
3.013 IU	54	47	87.0	4.0	(−9.1; 17.1)
587 IU	43	40	93.0	10.0	(−2.3; 22.2)
158 IU	37	31	83.8	0.7	(−14.5; 16.0)
31 IU	20	17	85.0	1.9	(−16.4; 20.3)

* P-value (Fisher’s exact test) = 0.700. SP: neutralizing antibodies ≥ 3.15 Log10 mIU/mL. Difference of SP: Difference of seropositivity (reduced dose – standard dose).

**Table 2:** Geometric mean antibody titers at 95 % CI (mIU/mL) by vaccine group.

Group	Total	GMT*	95 % CI	GMT Ratio	95 % CI
	N				
27.476 IU	59	3310.1	(2555.2; 4288.0)	−	−
10.447 IU	40	3748.3	(2786.0; 5042.9)	1.1	(0.9; 1.5)
3.013 IU	54	3508.0	(2723.4; 4518.6)	1.1	(0.8; 1.4)
587 IU	43	3969.4	(3132.7; 5029.6)	1.2	(0.9; 1.6)
158 IU	37	3464.5	(2631.3; 4561.5)	1.0	(0.8; 1.4)
31 IU	20	4427.9	(2859.8; 6856.0)	1.3	(1.0; 1.8)

* P-value (Kruskal-Wallis) = 0.896. GMT: Geometric mean antibody titers.

**Fig. 2:**
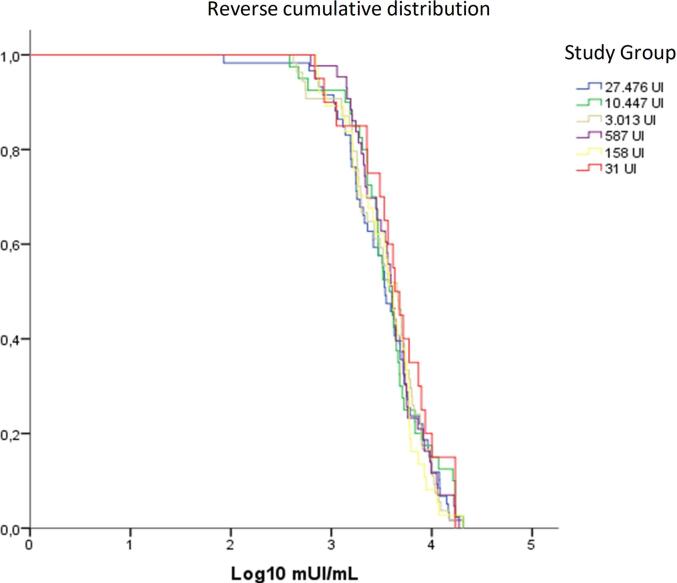
Reverse cumulative distribution of the level of neutralizing antibodies titers by vaccination group, 10 years after the initial vaccination.

The reverse cumulative distribution of neutralizing antibody titers shows similarity between groups (Fig. 2). A larger proportion of the 31 IU group exhibits higher value levels compared to the other groups.

Participants who did and those who did not live in or visit areas where vaccination against yellow fever was recommended showed similar seroconversion rates (p = 0.745; Table 3). Paradoxically, GMTs were substantially lower among participants who visited those areas, approaching statistically significance (Table 4).

**Table 3:** Serological status according to history of visit or residence in areas with recommendation of Yellow Fever vaccination.

Visit or resident in areas with recommendation for Yellow Fever vaccination	Seropositivity*				Total
	Yes		No		
	N	%	N	%	N
Yes	100	45.5	16	48.5	116
No	120	54.5	17	51.5	137
Total	220	100	33	100	253

* P-value (Chi-squared test) = 0.745. Participants with displacement to an unclassifiable area were considered as yes.

**Table 4:** Geometric mean titers of neutralizing antibodies and 95% confidence interval by displacement for area with recommendation for Yellow Fever vaccination.

Displacement for area with recommendation for Yellow Fever vaccination	N	GMT*	95 % CI
Yes	116	3277.7	2778.1–3867.2
No	137	3960.1	3404.5–4606.5

* P-value (Mann-Whitney test) = 0.074. Participants with displacement to an unclassifiable area were considered as yes. GMT: Geometric mean titers.

## 4 Discussion

The current study is a complementary investigation that evaluated the long-term duration of humoral immunity in a subset of volunteers originally receiving subdoses of 17DD-YF vaccine, enrolled in the dose–response study in 2009. It is also complementary to the study that assessed a segment of this cohort in 2017. The original 2009 cohort of the 749 participants results showed that doses down to 587 IU had similar immunogenicity to the full doses. Eight years later seropositivity was maintained in 85 % of 318 participants accessed and was similar across groups. Both studies supported the use of ≥ 587 IU for adequate and sustainable immune response. It was important to verify if this seropositivity observed after eight years was sustained following 10 years.

Evaluation of duration of immunity after yellow fever vaccination based on the available literature is a challenging task. Gotuzzo and colleagues did an extensive review identifying 8 studies which evaluated duration of immunity ≥ 10 years after vaccination, and the seropositivity rate ranged from 74,5% to 100 %. However, studies involving travelers to endemic regions cannot ensure that ¨natural booster¨ did not account for immunity after very long periods [5]. Kling and colleagues performed a systematic literature review and meta-analysis that included investigation of the duration of vaccine-induced protection with stratification according to the follow-up and included periods of five years up to more than 20 years. As a result in adults from both endemic and nonendemic regions, seroprotection rates were 88 % in outcomes above 5 years up to 10 years and 71 % for healthy adults in outcomes above 10 years up to 20 years (3 studies from endemic countries and one from non-endemic country) [8]. Jenny L. Schnyder and colleagues in a systematic review and meta-analysis regarding literature of long-term protection equal or above 10 years with findings seroprotection rates of 94 % in healthy adults in non-endemic settings and 76 % in endemic settings (all in Brazil) which was partly explained by higher cutoff seroprotection [9].

A review in 2019 by Roukens and Visser about the fractional-dose yellow fever vaccination that mentioned the 2009 and 2017 Bio-Manguinhos studies indicated that in healthy young volunteers, the protective response persists for 10 years or longer if this response occurs shortly after vaccination [28].

The results found in this study with the reassessment ten years after vaccination against yellow fever in young male adults from the military, who had seroconverted in 2009, and have not been revaccinated adds new data of evaluation in immunological response of lower doses of the 17DD YF vaccine 10 years after the prime dose bringing comparable results with the previous reviews. Serological testing detected neutralizing antibodies in 83 % or more of the participants from all vaccine groups, regardless of history of visits to areas where the yellow fever vaccine was recommended. The differences in seropositivity between recipients of the reference vs. lower doses vaccines did not disclose a clear pattern. Seropositivity levels 10 years after vaccination were substantially lower than those achieved 30 days after vaccination but were roughly similar to those observed 8 years after vaccination.

Despite the sizable differences in GMT between some vaccine groups the reverse cumulative distribution of titers did not show meaningful patterns.

There was a marked decrease in GMT in all vaccine's groups compared to 2009 levels. However, comparability of results may be hampered by improvement introduced in laboratory methods after 2009. Moreover, comparability across vaccine groups may have hampered by the selection process, as participants in this study were not a probabilistic sample of the original randomized groups.

The group of 31 IU had inferior immunogenicity on the dose–response study in 2009 and the highest rate of primary failures on seroconversion. For this reason, it is the group with the lowest number of participants in 2017 and current study. One possible explanation for the higher immunogenicity of this group in both last evaluation is that participants remaining seropositive in the vaccine subgroups with the lowest doses constituted a small, selected group, who could be high seroconverts, and should not represent the typical immune response of the original subgroup enrolled in 2009.

The limitations of the study were the reduced number of reassessed participants of the initial 2009 study, with implicates that the results cannot be generalized and cannot be used alone to establish revaccination. In addition, possible wild infection with YF virus or other flavivirus could impact seroprotection.

## 5 Conclusions

The long-term immunogenicity of lower doses, down to 587 IU supports their utilization in settings of increased sudden demand. Doses lowest than 587 IU displayed lower immunogenicity in the primary seroconversion in the first 2009-Bio-Manguinhos study.

Lower doses appear to have the potential for adoption in regular vaccination, pending assessment in children aged 9–23 months, and testing vaccines from other substrains and manufacturers.

Just as for the full dose, waning of immunity with lower doses could strengthen the argument of booster dose in adults, especially during epidemics.

These conclusions do not apply necessarily to the yellow fever vaccines from the other producers, which were not tested in this study.

Dose-response studies are still required for its universal use in children less than 2 – years of age, pregnant women as well as immunocompromised patients because the particularities of their immune response.

## Funding sources

The study received financial support through a grant from the Welcome Trust (grant 215474/Z/19/Z) and did not obtain additional funding from any specific sources. The funding source had no role in study design; collection, analysis and interpretation of data; writing of the report; or in the decision to submit the article for publication.

## CRediT authorship contribution statement

**Clarice Monteiro Vianna:** Writing – review & editing, Writing – original draft, Visualization. **Tatiana Guimarães Noronha:** Writing – review & editing, Supervision, Project administration, Methodology, Formal analysis, Conceptualization. **Luiz Antonio Bastos Camacho:** Writing – review & editing, Supervision, Project administration, Methodology, Formal analysis, Conceptualization. **Raissa Coelho Andrade:** Writing – review & editing, Visualization. **Ricardo Cristiano de Souza Brum:** Writing – review & editing. **Eliane Matos dos Santos:** Conceptualization. **Daniele Fernandes Aguiar:** Conceptualization. **Maria Leticia Borges dos Santos:** Project administration. **Robson Leite de Souza Cruz:** Project administration. **Sheila Maria Barbosa de Lima:** Writing – review & editing. **Adriana de Souza Azevedo Soares:** Writing – review & editing. **Waleska Dias Schwarcz:** Writing – review & editing. **Thalita da Matta de Castro:** Validation, Software, Methodology, Formal analysis, Data curation, Conceptualization. **Janaína Reis Xavier:** Validation, Software, Methodology, Formal analysis, Data curation, Conceptualization. **Deborah Araújo da Conceição:** Project administration. **Akira Homma:** Investigation, Conceptualization. **Maria de Lourdes de Sousa Maia:** Resources, Funding acquisition.

## Declaration of competing interest

The authors declare that they have no known competing financial interests or personal relationships that could have appeared to influence the work reported in this paper.

## Data Availability

Data will be made available on request.
